# A real-world study of acute and preventive medication use, adherence, and persistence in patients prescribed fremanezumab in the United States

**DOI:** 10.1186/s10194-022-01413-z

**Published:** 2022-05-04

**Authors:** Lynda J. Krasenbaum, Vasantha L. Pedarla, Stephen F. Thompson, Krishna Tangirala, Joshua M. Cohen, Maurice T. Driessen

**Affiliations:** 1 Teva Pharmaceutical Industries, West Chester, PA USA; 2 STATinMED Research, Ann Arbor, MI USA; 3 Teva Pharmaceutical Industries, Parsippany, NJ USA; 4 Teva Pharmaceutical Industries, Amsterdam, The Netherlands

**Keywords:** Fremanezumab, Migraine, Prevention, Real-world data, Adherence, Persistence, Comorbidity

## Abstract

**Background:**

Following approval of fremanezumab for the prevention of migraine in adults, health care decision makers are interested in understanding real-world clinical characteristics and treatment patterns among patients initiating fremanezumab therapy.

**Methods:**

Data were obtained for this retrospective (pre-post) study from the Veradigm Health Insights database. The study period was January 1, 2014, to June 30, 2019. Patients were included if they were aged ≥ 18 years; had ≥ 1 migraine diagnosis during the study period; and had a medication record for fremanezumab on or after diagnosis during the identification period (September 1, 2018–December 31, 2018). Treatment patterns, including adherence, persistence, and utilization of acute and preventive migraine medication prescriptions, were evaluated.

**Results:**

Of 987 patients initiating fremanezumab during the study period, 738 (74.8%) were adherent to fremanezumab by proportion of days covered (PDC; ≥ 80%) and 780 (79.0%) were adherent by medication possession ratio (MPR; ≥ 80%). A total of 746 (75.6%) patients were persistent for ≥ 6 months. Quarterly fremanezumab (*n* = 186) was associated with higher rates of adherence versus monthly fremanezumab (*n* = 801) by PDC (quarterly, 91.3%; monthly, 84.9%; *P* < 0.001) and MPR (quarterly, 92.2%; monthly, 87.9%; *P* = 0.006) and higher persistence at ≥ 6 months (quarterly, 82.8%; monthly, 73.9%; *P* = 0.011). After fremanezumab initiation, patients who were persistent for ≥ 6 months experienced significant reductions from baseline in the mean monthly number of acute and preventive migraine medication prescriptions (*P* < 0.001). Subgroup analyses in patients with comorbid depression and anxiety showed meaningful real-world benefits based on significant reductions in the number of patients who were prescribed antidepressants (baseline, 68.6%; follow-up, 56.4%; *P* = 0.0025) and anxiolytic medications (baseline, 55.0%; follow-up, 47.2%; *P* = 0.037), respectively. In a subgroup of patients with comorbid hypertension at baseline, fremanezumab treatment resulted in nonsignificant reductions in blood pressure.

**Conclusions:**

Overall, adherence and persistence to fremanezumab in this real-world study was high in patients with migraine, with higher rates observed for quarterly fremanezumab. Patients who were persistent for ≥ 6 months experienced significant reductions in acute and preventive migraine medication use, while a subgroup of migraine patients with comorbid depression and anxiety at baseline showed significant reductions in antidepressant and anxiolytic medication use.

**Supplementary Information:**

The online version contains supplementary material available at 10.1186/s10194-022-01413-z.

## Background

Fremanezumab is a fully humanized monoclonal antibody (IgG2Δa) that selectively targets calcitonin gene-related peptide (CGRP) [1], a 37 amino acid neuropeptide that is expressed widely in the central and peripheral nervous system and has been implicated in migraine pathophysiology [2]. Migraine is the second most prevalent cause of disability, as defined by years lived with disability [3]. Migraine is broadly classified by frequency as either episodic migraine (EM; < 14 migraine days per month) or chronic migraine (CM; ≥ 15 migraine days per month for > 3 months) [4].

Fremanezumab has been found to be effective and well tolerated across a wide patient population in 3 randomized, double-blind, phase 3 clinical trials, including the 12-week HALO EM (*n* = 875 [included patients with EM]) [5], the 12-week HALO CM (*n* = 1130 [included patients with CM]) [6] studies, the 12-month HALO long-term extension study (*n* = 1890 [included both roll-over patients from HALO EM and HALO CM and new patients]) [7], and the 12-week FOCUS study (*n* = 838 [included patients with EM or CM and inadequate response to 2–4 prior migraine preventive treatment classes]) [8]. Based on the pivotal HALO EM and HALO CM studies, fremanezumab was approved by the US Food and Drug Administration as a subcutaneous formulation administered quarterly (675 mg every 3 months) or monthly (225 mg monthly) for the preventive treatment of migraine in adults [9].

Following the clinical investigation and regulatory approval of fremanezumab, it is valuable to have real-world data on treatment adherence, persistence, and changes in acute medication use with monoclonal antibody therapies targeting the CGRP pathway. The clinical practice in the real-world may substantially vary from controlled trials. In this context, real-world data not only provides an important addition to the body of evidence on a specific treatment but may also inform policy makers and drive updates to clinical guidelines. The purpose of this US real-world study is to describe the patient demographics and clinical characteristics, and to assess adherence, persistence, and impact on acute and preventive migraine medication prescription use among patients with migraine who initiated quarterly or monthly fremanezumab therapy.

## Methods

### Data sources

**Fig. 1:**
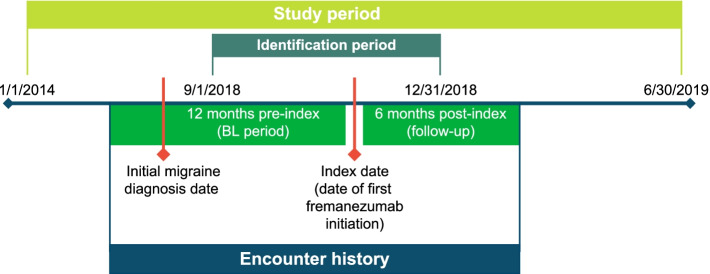
Overview of study design. *BL* baseline. *Note*. The first migraine diagnosis date during the 12-month baseline period was considered the initial diagnosis date

The study period was from January 1, 2014, to June 30, 2019 (Fig. 1). Data for this retrospective (pre-post) study was drawn from the Veradigm Health Insights database (a combination of Allscripts and Practice fusion). The Veradigm Health Insights database consists of data collected through the Allscripts cloud-based ambulatory electronic health records (EHRs), as well as Allscripts-hosted and on-premises ambulatory EHR platforms, representing data from over 120,000 providers in all 50 US states. Key data elements captured by the Allscripts systems include demographics, family history, prescription data (national drug codes), diagnosis data (*International Classification of Diseases, Ninth/Tenth Revisions, Clinical Modifications* [ICD-9/10-CM] codes), laboratory test orders and results, physician characteristics, office visits, and vital signs. Data are made available for analysis in a de-identified research database, compliant with the Health Insurance Portability and Accountability Act (HIPAA) of 1996.

### Patient eligibility

Patients were included in the study population if they had ≥ 1 migraine diagnosis occurring during the study period (the first migraine diagnosis date during the 12-month baseline period was considered the initial diagnosis date), had a medication record for fremanezumab on or after the initial diagnosis date during the identification period (September 1, 2018–December 31, 2018; fremanezumab initiation date was considered the index date; Fig. 1), and were aged ≥ 18 years on the index date. Patients were excluded from the study population if records contained missing/unknown age or sex information, evidence of CGRP pathway–targeted therapy prior to the index date, or evidence of pregnancy or childbirth at any time during the study period.

### Study outcomes

Adherence was measured by the proportion of days covered (PDC) and medication possession ratio (MPR). PDC was defined as the number of days covered by fremanezumab divided by a fixed follow-up period of 6 months. This approach provides a conservative measure of adherence because it does not double count for overlap due to early refills. MPR was defined as the sum of the days of supply for fremanezumab divided by a fixed follow-up period of 6 months. The MPR calculation assumes that if a patient fills the prescription early, they would not start to take the new prescription until they completed the expected days of supply from the previous prescription [10]; however, use of fremanezumab as prescribed could not be assessed based on the available data. Patients were classified as high adherers if they had a PDC ≥ 80% or MPR ≥ 80% [11]. Persistence with fremanezumab was measured as the number of days from the index date until the earliest discontinuation of fremanezumab or the end of follow-up, including persistence for ≥ 3 months and ≥ 6 months. Adherence and persistence were evaluated for all patients with dosing data. Treatment discontinuation was defined as a gap of > 45 days between 2 consecutive prescription orders for fremanezumab followed by activity in the EHR. The number of patients who discontinued treatment, switched treatment (indicated by any acute/preventive medication after discontinuation), and reinitiated treatment (restarted fremanezumab therapy after a > 45-day gap) for their first treatment change were also evaluated. Changes in acute medication (triptans, ergot alkaloids, nonsteroidal anti-inflammatory drug [NSAID], analgesics, and opioid analgesics) and preventive medication (anticonvulsants/antiepileptics, antihypertensives, muscle relaxants, antidepressants, antihistamines, and onabotulinumtoxinA) prescription use and the total number of prescriptions following fremanezumab initiation were examined. Additionally, subgroup analyses were performed to examine outcomes for patients with the most common comorbidities: depression (changes in antidepressant medication utilization), anxiety (changes in anxiolytic medication utilization), and hypertension (changes in recorded systolic blood pressure [SBP] and diastolic blood pressure [DBP]) as reported in EHR.

### Statistical analyses

All study variables were first examined descriptively. Dichotomous and polychotomous variables were described by number and percentage, and continuous variables were described by mean and standard deviation (SD). Statistical tests of significance for differences in the baseline characteristics among patients initiating quarterly and monthly fremanezumab were calculated via chi-square test for dichotomous variables and student t-test for continuous variables. Statistical tests of the significance of the difference in the distributions between the 6-month baseline and follow-up periods were calculated via McNemar’s test for dichotomous variables and the paired t-test or Wilcoxon signed rank test for continuous variables. A *P-*value of < 0.05 was considered statistically significant. Statistical analyses were performed using SAS version 9.4 (Cary, NC).

## Results

### Patient characteristics

In total, 3716 patients with a prescription for fremanezumab were identified. Of those, 1263 had data available for the 6 months after first fremanezumab initiation, and 987 had a known fremanezumab dose at the index date (quarterly, *n* = 186; monthly, *n* = 801). Of the 987 patients in the study population, 60.2% (*n* = 594) of patients had EM and 39.8% (*n* = 393) of patients had CM. Baseline characteristics and migraine-specific common comorbidities are summarized in Table 1. The majority of patients identified were female (*n* = 842 [85.3%]), and depression (*n* = 143 [14.5%]), anxiety (*n* = 152 [15.4%]), and hypertension (*n* = 115 [11.7%]) were among the most common comorbidities. Demographics and baseline characteristics were generally balanced between the patients receiving quarterly and monthly dosing of fremanezumab.

**Table 1:** Baseline patient characteristics

		Defined dose		
	Overall (*N* = 987)	Quarterly (*n* = 186)	Monthly (*n* = 801)	*P* value^a^ (quarterly vs monthly)
Age, years, mean (SD)	46.8 (12.8)	47.1 (13.6)	46.7 (12.6)	0.697
Age, years, n (%)				
18–40	314 (31.8)	59 (31.7)	255 (31.8)	0.976
41–64	595 (60.3)	104 (55.9)	491 (61.3)	0.176
≥ 65	78 (7.9)	23 (12.4)	55 (6.9)	0.012
Sex, n (%)				
Female	842 (85.3)	161 (86.6)	681 (85.0)	0.593
Male	145 (14.7)	25 (13.4)	120 (15.0)	0.593
Migraine diagnosis, n (%)				
EM	594 (60.2)	123 (66.1)	471 (58.8)	
CM	393 (39.8)	63 (33.9)	330 (41.2)	
Time from diagnosis to initial treatment, months, mean (SD)	29.2 (20.8)	27.1 (21.1)	29.6 (20.8)	0.141
Provider specialty, n (%)				
Neurology	526 (53.3)	82 (44.0)	444 (55.4)	0.005
Family medicine	122 (12.4)	30 (16.1)	92 (11.5)	0.083
Psychiatry	93 (9.4)	32 (17.2)	61 (7.6)	< 0.001
Specialist/nurse/PA	24 (2.4)	5 (2.7)	19 (2.4)	0.801
Physical medicine	19 (1.9)	0	19 (2.4)	N/A
Anesthesiology/pain management	13 (1.3)	8 (4.3)	5 (0.6)	< 0.001
Headache orofacial pain	15 (1.5)	0	15 (1.9)	N/A
Other^b^	175 (17.7)	29 (15.6)	146 (18.2)	0.396
Quan-Charlson comorbidity index score, mean (SD)	0.30 (0.84)	0.32 (0.99)	0.29 (0.80)	0.684
0, n (%)	813 (82.4)	154 (82.8)	659 (82.3)	0.688
1–3	161 (16.3)	28 (15.1)	133 (16.6)	0.764
4–6	10 (1.0)	3 (1.6)	7 (0.9)	0.240
> 6	3 (0.3)	1 (0.5)	2 (0.2)	0.113
Common (> 10%) migraine-specific comorbidities, n (%)				
Anxiety (including GAD)	152 (15.4)	30 (16.1)	122 (15.2)	0.760
Depression (including MDD)	143 (14.5)	32 (17.2)	111 (13.9)	0.243
High cholesterol	118 (12.0)	23 (12.4)	95 (11.9)	0.848
Back pain	117 (11.9)	20 (10.8)	97 (12.1)	0.606
Hypertension	115 (11.7)	24 (12.9)	91 (11.4)	0.555
Neck pain	102 (10.3)	25 (13.4)	77 (9.6)	0.122
Insomnia	100 (10.1)	26 (14.0)	74 (9.2)	0.054

*SD* standard deviation, CM chronic migraine, EM episodic migraine, *GAD* generalized anxiety disorder, *MDD* major depressive disorder

^a^*P* values calculated via chi-square test for dichotomous variables or Fisher’s exact test for continuous variables

^b^Other includes specialist, allergy/immunology, other, or missing

### Adherence and persistence to fremanezumab treatment

Overall, adherence to fremanezumab treatment was high, with a mean PDC of 86.1% (SD, 24.3%) and a mean MPR of 88.7% (SD, 23.0%) at 6 months after fremanezumab initiation. A total of 738 (74.8%) and 780 (79.0%) patients were classified as higher adherers by PDC (≥ 80%) and MPR (≥ 80%), respectively. The mean persistence duration was 157.5 (SD, 46.5) days, with 814 (82.5%) patients persistent for ≥ 3 months and 746 (75.6%) patients persistent for ≥ 6 months. A total of 216 (21.9%) patients discontinued fremanezumab treatment within 6 months after first initiation, with 99 (45.8%) patients switching to an alternative preventive medication, of which 95 (44.0%) patients switched to a different class of preventive medication (Table 2).

**Table 2:** Discontinuation rates of fremanezumab and subsequent medication changes

	Overall (*N* = 216)	Quarterly (*n* = 29)	Monthly (*n* = 187)
Discontinuation, n (%)	216 (100.0)	29 (100.0)	187 (100.0)
Switching, n (%)	99 (45.8)	13 (44.8)	86 (46.0)
Within-class switching	17 (7.9)	4 (13.8)	13 (7.0)
Aimovig	11 (5.1)	1 (3.4)	10 (5.3)
Emgality	7 (3.2)	3 (10.3)	4 (2.1)
Between-class switching	95 (44.0)	12 (41.4)	83 (44.4)
Acute medications	46 (21.3)	5 (17.2)	41 (21.9)
Triptans	19 (8.8)	3 (10.3)	16 (8.6)
Ergot alkaloids	1 (0.5)	0	1 (0.5)
NSAID analgesics	19 (8.8)	2 (6.9)	17 (9.1)
Opioid analgesics	12 (5.6)	1 (3.4)	11 (5.9)
Miscellaneous	7 (3.2)	1 (3.4)	6 (3.2)
Preventive medication	81 (37.5)	10 (34.5)	71 (38.0)
Anticonvulsants/antiepileptics	26 (12.0)	4 (13.8)	22 (11.8)
Antihypertensive	19 (8.8)	1 (3.4)	18 (9.6)
Muscle relaxants	16 (7.4)	3 (10.3)	13 (7.0)
Antidepressants	27 (12.5)	1 (3.4)	26 (13.9)
Antihistamines	1 (0.5)	0	1 (0.5)
OnabotulinumtoxinA	7 (3.2)	0	7 (3.7)
Reinitiation, n (%)			
Any dose	28 (13.0)	2 (6.9)	26 (13.9)
Monthly to quarterly	1 (0.5)	0	1 (0.5)
Quarterly to monthly	0	0	0
Permanent discontinuation, n (%)	103 (47.7)	14 (48.3)	89 (47.6)

*NSAID* nonsteroidal anti-inflammatory drug

**Fig. 2:**
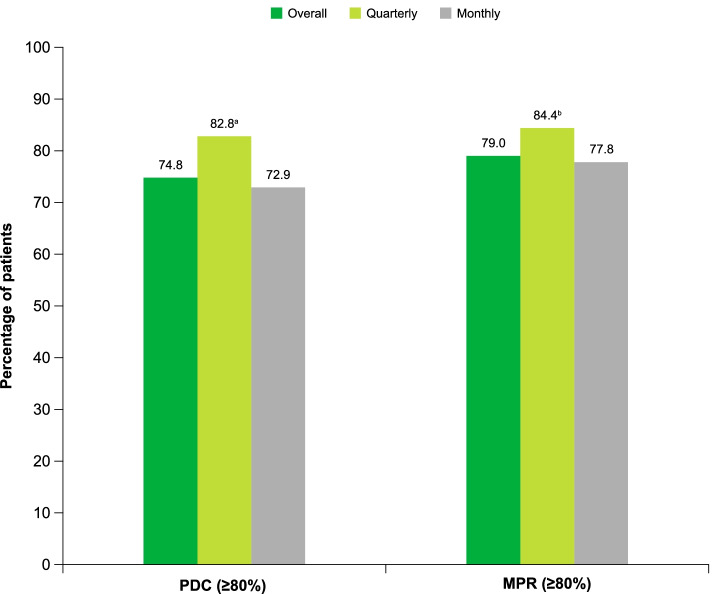
Adherence to fremanezumab therapy. *PDC* proportion of days covered; *MPR* medication possession ratio. *P* values calculated between quarterly dosing group and monthly dosing group via independent t-test. ^a^*P* = 0.005. ^b^*P* = 0.045

Further, the mean PDC and MPR of monthly fremanezumab was 84.9% (SD, 25.3%) and 87.9% (SD, 23.9%), respectively. In total, 584 (72.9%) patients were classified as higher adherers by PDC (≥ 80%) and 623 (77.8%) patients by MPR (≥ 80%). In comparison, the mean PDC and MPR of quarterly fremanezumab was 91.3% (SD, 18.3%) and 92.2% (SD, 18.2%), respectively, with 154 (82.8%) patients classified as higher adherers by PDC (≥ 80%) and 157 (84.4%) patients by MPR (≥ 80%; Fig. 2). Adherence was statistically significantly higher among patients receiving quarterly fremanezumab versus monthly fremanezumab therapy at 6 months (*P* ≤ 0.006). Similarly, mean persistence rate was statistically significantly higher among patients receiving quarterly fremanezumab versus monthly fremanezumab therapy (*P* = 0.011). Patients discontinued quarterly fremanezumab therapy at a lower rate than monthly fremanezumab (quarterly dosing, 15.6% [*n* = 29]; monthly dosing, 23.3% [*n* = 187]; *P* = 0.021; Table 2).

### Acute and preventive medication use

For the 746 patients classified as persistent for ≥ 6 months, significant reductions in the mean number of acute migraine medications per month were observed for NSAIDs (baseline, 0.39 [SD, 0.95]; follow-up, 0.30 [SD, 0.78]; *P* = 0.010). No significant changes were observed in the mean number of all acute migraine prescriptions per month (baseline, 1.89 [SD, 2.86]; follow-up, 1.87 [SD, 2.65]; *P* = 0.816). Among preventive medications used, a significant reduction was observed in the mean number of antidepressant medications per month (baseline, 0.62 [SD, 1.21]; follow-up, 0.48 [SD, 1.02]; *P* = 0.002; Table 3).

**Table 3:** Acute and preventive medication prescription trends among persistent (≥ 6 months) patients with a defined dose (*n* = 746)

	Number of prescriptions/month		
	Baseline	Follow-up	*P* value^a^
Acute medication, mean (SD)	1.89 (2.86)	1.87 (2.65)	0.816
Triptans	0.68 (1.12)	0.76 (1.18)	0.111
Ergot alkaloids	0.04 (0.41)	0.04 (0.38)	0.887
NSAID analgesics	0.39 (0.95)	0.30 (0.78)	***0.010***
Opioid analgesics	0.56 (2.06)	0.49 (1.72)	0.246
Miscellaneous	0.21 (0.73)	0.28 (1.20)	0.071
Preventive nonspecific migraine medication, mean (SD)	2.22 (2.62)	2.14 (2.48)	0.349
Anticonvulsants/antiepileptics	0.81 (1.36)	0.82 (1.40)	0.823
Antihypertensive	0.34 (0.82)	0.41 (0.99)	0.056
Muscle relaxants	0.31 (0.90)	0.30 (0.81)	0.820
Antidepressants	0.62 (1.21)	0.48 (1.02)	***0.002***
Antihistamines	0.01 (0.12)	0.01 (0.12)	0.655
OnabotulinumtoxinA	0.14 (0.52)	0.12 (0.55)	0.315

*SD* standard deviation, *NSAID* nonsteroidal anti-inflammatory drug

^a^*P* values calculated via paired t-test; bold, italicized *P* values indicate statistical significance

**Fig. 3:**
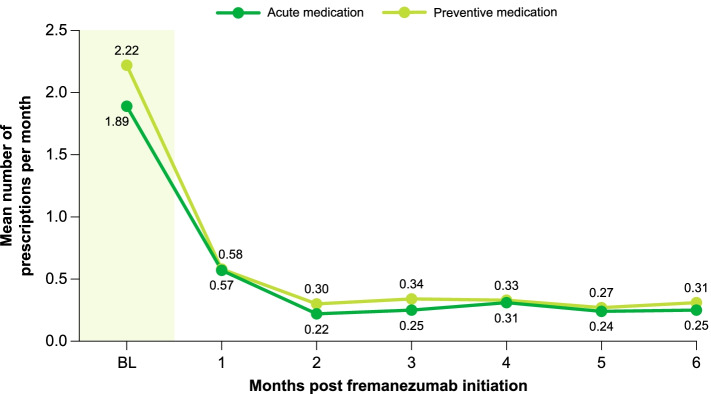
Number of acute medication prescriptions analyzed via individual month post-fremanezumab initiation. *BL* baseline. *P* values calculated between individual months and baseline values via paired t-test. ^a^*P* < 0.001

When further analyzed by individual month post-initiation, a significant reduction was observed in both the mean number of acute medication prescriptions per month at each monthly time point (all *P* < 0.001) and the mean number of preventive nonspecific migraine medications per month (all *P* < 0.001; Fig. 3). Significant decreases were observed in each category of acute (triptans, ergot alkaloids, NSAID analgesics, opioid analgesics) and preventive (anticonvulsants/antiepileptics, antihypertensives, antidepressants, onabotulinumtoxinA) migraine prescriptions evaluated when analyzed monthly and compared with the mean 6-month baseline period (Table 4).

**Table 4:** Mean monthly number of medication prescriptions among persistent (≥ 6 months) patients with a defined dose (*n* = 746)

	Baseline	Months post-initiation					
		1	2	3	4	5	6
Acute medication, mean (SD)	1.89 (2.86)	0.57 (0.91)^a^	0.22 (0.69)^a^	0.25 (0.61)^a^	0.31 (1.04)^a^	0.24 (0.67)^a^	0.25 (0.74)^a^
Triptans	0.68 (1.12)	0.30 (0.60)^a^	0.08 (0.36)^a^	0.09 (0.35)^a^	0.11 (0.37)^a^	0.10 (0.41)^a^	0.08 (0.29)^a^
Ergot alkaloids	0.04 (0.41)	0.01 (0.10)^c^	0.01 (0.14)^b^	0.004 (0.08)^b^	0.01 (0.10)^b^	0.01 (0.10)^c^	0.01 (0.07)^b^
NSAID analgesics	0.39 (0.95)	0.08 (0.33)^a^	0.03 (0.18)^a^	0.04 (0.21)^a^	0.05 (0.25)^a^	0.03 (0.17)^a^	0.05 (0.32)^a^
Opioid analgesics	0.56 (2.06)	0.10 (0.49)^a^	0.07 (0.34)^a^	0.07 (0.31)^a^	0.08 (0.58)^a^	0.06 (0.39)^a^	0.08 (0.42)^a^
Preventive nonspecific migraine medication, mean (SD)	2.22 (2.62)	0.58 (0.94)^a^	0.30 (0.66)^a^	0.34 (0.77)^a^	0.33 (0.78)^a^	0.27 (0.62)^a^	0.31 (0.70)^a^
Anticonvulsants/antiepileptics	0.81 (1.36)	0.25 (0.56)^a^	0.10 (0.35)^a^	0.11 (0.41)^a^	0.15 (0.57)^a^	0.10 (0.39)^a^	0.12 (0.40)^a^
Antihypertensive	0.34 (0.82)	0.10 (0.35)^a^	0.07 (0.30)^a^	0.06 (0.28)^a^	0.07 (0.30)^a^	0.05 (0.27)^a^	0.06 (0.29)^a^
Antidepressants	0.62 (1.21)	0.12 (0.37)^a^	0.07 (0.30)^a^	0.09 (0.39)^a^	0.06 (0.27)^a^	0.06 (0.27)^a^	0.07 (0.29)^a^
Botox	0.14 (0.52)	0.03 (0.18)^a^	0.02 (0.14)^a^	0.03 (0.20)^a^	0.01 (0.10)^a^	0.01 (0.11)^a^	0.02 (0.18)^a^

*SD* standard deviation, *NSAID* nonsteroidal anti-inflammatory drug

*P* values calculated via paired t-test against baseline mean

^a^*P* < 0.001

^b^*P* = 0.005

^c^*P* = 0.023

### Subgroup analyses: migraine with comorbid depression

The comorbid depression subgroup (*n* = 172) had a mean age of 46.7 (SD, 13.5) years and registered a mean Quan-Charlson comorbidity index score of 0.63 (SD, 1.15), both relatively comparable to the overall population (Table S1). Among patients with comorbid depression, the total number of patients with antidepressant medication prescriptions significantly decreased (baseline, 118 [68.6%]; follow-up, 97 [56.4%]; *P* = 0.003; Table 5).

**Table 5:** Subgroup analysis by presence of comorbid depression, anxiety, and hypertension

	Patients with migraine and comorbid depression cohort (*n* = 172)		
	Baseline	Follow-up	*P* value
Total number of patients with antidepressant prescriptions, n (%)	118 (68.6)	97 (56.4)	***0.003***
Total number of antidepressant prescriptions, mean (SD)	1.05 (0.96)	0.84 (0.95)	***0.008***
	**Patients with migraine and comorbid anxiety cohort (*n*** **= 180)**		
	**Baseline**	**Follow-up**	***P*** **value**
Total number of patients with anxiolytic prescriptions, n (%)	99 (55.0)	85 (47.2)	***0.037***
Total number of anxiolytic prescriptions, mean (SD)	0.75 (0.88)	0.66 (0.81)	0.182
	**Patients with migraine and comorbid hypertension cohort (*n*** **= 142)**		
	**Baseline**	**Follow-up**	***P*** **value**
SBP, mean (SD)	127.3 (15.0)	127.0 (14.6)	0.837
DBP, mean (SD)	78.4 (10.0)	77.8 (10.6)	0.562

*SD standard deviation, SBP* systolic blood pressure, *DBP* diastolic blood pressure

*P* values calculated via McNemar’s test and the paired t-test or Wilcoxon signed rank test to dichotomous and continuous variables, respectively; bold, italicized *P* values indicate statistical significance

### Subgroup analyses: migraine with comorbid anxiety

In the comorbid anxiety subgroup (*n* = 180), both mean age (44.7 [SD, 13.3] years) and mean Quan-Charlson comorbidity index score (0.49 [SD, 0.93]) were relatively comparable to the overall population (Table S1). The total number of patients with anxiolytic medication prescriptions significantly decreased among persistent patients with comorbid anxiety (baseline, 99 [55.0%] patients; follow-up, 85 [47.2%], *P* = 0.037; Table 5).

### Subgroup analyses: migraine with comorbid hypertension

Patients in the comorbid hypertension subgroup (*n* = 142) trended towards a higher mean age (54.0 [SD, 12.0] years) than the overall population. Additionally, the mean Quan-Charlson comorbidity index score in the comorbid hypertension subgroup trended higher (0.92 [SD, 1.32]) than in the overall population (Table S1). Nonsignificant changes were observed for patients with comorbid hypertension in mean SBP (baseline, 127.3 [SD, 15.0]; follow-up, 127.0 [SD, 14.6]; *P* = 0.8374) and DBP (baseline, 78.4 [SD, 10.0]; follow-up, 77.8 [SD, 10.6]; *P* = 0.562; Table 5).

## Discussion

In this real-world study, patients with migraine who initiated fremanezumab as preventive treatment showed high adherence and persistence; both adherence and persistence rates were higher among patients receiving quarterly fremanezumab versus monthly fremanezumab treatment. Statistically significant reductions in acute and preventive medication use were observed among patients who were persistent with fremanezumab treatment. A similar pattern of benefit in terms of reductions in antidepressant use and anxiolytic use was observed in subgroups of migraine patients with comorbid depression and anxiety.

Oral migraine medication adherence (defined as the extent to which a patient follows directions with regard to the prescribed interval and dose of a dosing regimen) and persistence (defined as the duration of time from initiation to discontinuation of therapy) have been variable and low. In a systematic review of oral migraine preventive adherence and persistence, treatment adherence in observational studies ranged from 21% to 80% at 6 months, while treatment persistence ranged from 19% to 79% at 6 months, and pooled persistence from randomized controlled trials on propranolol, amitriptyline, and topiramate showed rates of 77%, 55%, and 57%, respectively, at 16 to 26 weeks [12]. Further, low adherence rates have been linked with inferior clinical outcomes and higher health care costs [13]. The class of CGRP pathway–targeted migraine preventive therapeutics was developed, in part, to address low adherence rates to oral migraine preventive medications. In a recently conducted retrospective claims database study, patients who received monthly erenumab showed adherence rates at 6 months ranging from 31% (PDC ≥ 0.80) to 42% (MPR ≥ 0.80) [14]. Resolution of migraine symptoms and effectiveness of treatment have been shown to impact patient adherence [15]. Further, persistence is influenced by several patient and treatment factors; lack of efficacy and side effects are the 2 largest drivers of discontinuation of oral migraine preventive medications [16]. In this context, results from our study are notable as about 75% to 80% of patients were adherent to fremanezumab therapy at 6 months, with statistically significantly higher adherence with quarterly dosing versus monthly dosing. Previous studies have shown that flexible dosing strategies, availability of treatment choices, and physician/patient communication can promote treatment adherence [17–19]. Patient preference of dosing regimens may confer a sense of control over treatment choice and can help drive treatment adherence [18]. Our results confirm that treatment adherence was slightly better for quarterly versus monthly fremanezumab. These results may be linked to patient preference, as observed in previous studies wherein more patients with migraine preferred fremanezumab quarterly dosing over monthly dosing [18]. Data captured in the current study represent only prefilled syringe administration prescriptions. To this end, there is a potential for further improvement in fremanezumab adherence and persistence rates by the increasing adoption of an autoinjector/prefilled pen route of administration.

Medication overuse among patients with migraine can lead to a poorer quality of life and higher economic costs [20, 21]; reducing concomitant medication usage, especially with acute medications (which can be considered as a proxy for monthly migraine days) is an important goal in a preventive migraine therapeutic strategy. Among patients who were persistent with fremanezumab treatment, acute migraine medication prescription use significantly decreased during the 6-month follow-up period. Previously, a post hoc subgroup analysis of the HALO CM study found that a higher proportion of patients reverted from medication overuse to no medication overuse after 12 weeks of fremanezumab treatment (quarterly fremanezumab, 55.2% [*P* = 0.0389]; monthly fremanezumab, 60.6% [*P* = 0.0024]; vs placebo, 46.3%) [22]. Further, results from the FOCUS study showed that fremanezumab treatment for 12 weeks led to a reduction in the monthly average number of days of use of any acute migraine medication when compared to placebo (least square mean difference: quarterly fremanezumab, –3.1 [*P* < 0.0001]; monthly fremanezumab, –3.4 [*P* < 0.0001]) [8]. The findings from the current real-world study are similar to those observed in clinical trials and contribute to the growing body of evidence on fremanezumab treatment and the reduction in concomitant medication utilization.

In addition to medication overuse, comorbidities including depression and anxiety have been shown to be associated with migraine [23] and are known to adversely impact quality of life and increase health care resource utilization and costs. [24, 25]. In a post hoc subgroup analysis of the HALO CM study, patients with CM and comorbid depression treated with fremanezumab showed significant reductions in monthly migraine days and depression burden [26]. Results from this real-world study also showed significant reductions in prescribed antidepressant and anxiolytic medication use in migraine patients with comorbid depression and anxiety, respectively. Taken together, the results of the current real-world evidence study confirms that fremanezumab offers clinically meaningful benefits in these subpopulations with difficult-to-treat migraine.

There have been speculative concerns regarding potential cardiovascular side effects associated with CGRP pathway-targeted treatments, as CGRP may act as a vasodilatory safeguard [27]; however, no serious cardiovascular adverse events were reported from clinical investigations of CGRP pathway–targeted treatments [28]. Recently, studies evaluating the safety profile of erenumab in the post-market setting have reported elevated blood pressure [29, 30]. In light of these findings, erenumab prescribing information was amended to include the association of risk of hypertension with erenumab use [31]. We examined the blood pressure changes among patients with comorbid hypertension to investigate if a similar phenomenon to erenumab was present for fremanezumab. In our real-world study, we found slight reductions (although nonsignificant) in blood pressure in a subgroup of patients with comorbid hypertension after fremanezumab treatment.

The strengths of this study include its real-world setting and the use of the largest de-identified ambulatory EHR database that includes data from Allscripts and Practice Fusion for a broad, geographically diverse population that allowed for evaluation of both patient-level data and validated prescription data. In addition, a relatively high number of patients who had initiated fremanezumab had 6 months of available follow-up data. This study has a few limitations. The retrospective database-based design limited our ability to analyze additional outcomes of interest, such as patient-reported outcomes. Data on patient treatment reinitiation could not be obtained after the 6-month follow-up period. Although adherence and persistence were assessed in the current study, no information could be obtained from the database on whether patients were taking fremanezumab as prescribed by their physician [13]. Finally, Veradigm captures health data from the US population, limiting the generalizability of this study to this population. Additional studies would be needed to support these findings in patients with migraine in other US populations outside the Veradigm database or in other countries.

Overall, in this real-world study, patients with migraine who received fremanezumab treatment showed high adherence and persistence, with reductions in acute and preventive migraine drug utilization. Quarterly dosing of fremanezumab showed significantly higher adherence and persistence when compared to monthly fremanezumab. Additionally, fremanezumab treatment had meaningful real-world benefits for patients with comorbid depression and anxiety, as quantified by antidepressant and anxiolytic prescription utilization, and patients with migraine with comorbid hypertension showed no increase in SBP or DBP. Altogether, these results show that fremanezumab provides an effective and well-tolerated treatment option in US real-world clinical practice. Reducing acute and preventive medication utilization could decrease the overall medication burden for patients with migraine, including those with comorbid psychiatric disorders, such as depression and anxiety. The results of the current study may aid in clinical decision making regarding treatment choice and optimal dosing regimen, including in patients with psychiatric comorbidities.

## Supplementary Information

**Additional file 1.**

## Data Availability

The data that support the findings of this study are available from the corresponding author upon reasonable request.

## Abbreviations

CGRP: Calcitonin gene-related peptide
CM: Chronic migraine
DPB: Diastolic blood pressure
EHR: Electronic health record
EM: Episodic migraine
GAD: Generalized anxiety disorder
HIPAA: Health Insurance Portability and Accountability Act
ICD-9-CM: *International Classification of Diseases, Ninth Revision, Clinical Modification*
ICD-10-CM: *International Classification of Diseases, Tenth Revision, Clinical Modification*
MDD: Major depressive disorder
MRP: Medication possession ratio
NSAID: Nonsteroidal anti-inflammatory drug
PDC: Proportion of days covered
SBP: Systolic blood pressure
SD: Standard deviation
