# Supplementary material for: A real-world study of acute and preventive medication use, adherence, and persistence in patients prescribed fremanezumab in the United States
Source: J Headache Pain. 2022 May 4;23(1):54. doi: 10.1186/s10194-022-01413-z (PMC9066733; doi:10.1186/s10194-022-01413-z)
Supplement: Supplementary file 1 — Additional file 1. [file 10194_2022_1413_MOESM1_ESM.docx]

# Supplementary Material

# Table S1. Baseline patient characteristics among comorbidity subgroups

|  | Patients with migraine and comorbid depression (n = 172) | Patients with migraine and comorbid anxiety  (n = 180) | Patients with migraine and comorbid hypertension  (n = 142) |
| --- | --- | --- | --- |
| Age, years, mean (SD) | 46.7 (13.5) | 44.7 (13.3) | 54.0 (12.0) |
| Age, years, n (%) |  |  |  |
| 18-40 | 51 (29.7) | 71 (39.4) | 22 (15.5) |
| 41-64 | 105 (61.0) | 92 (51.1) | 92 (64.8) |
| ≥65 | 16 (9.3) | 17 (9.4) | 28 (19.7) |
| Sex, n (%) |  |  |  |
| Female | 149 (86.6) | 156 (86.7) | 108 (76.1) |
| Male | 23 (13.4) | 24 (13.3) | 34 (23.9) |
| Migraine diagnosis, n (%) |  |  |  |
| Episodic migraine | 103 (59.9) | 118 (65.6) | 94 (66.2) |
| Chronic migraine | 69 (40.1) | 62 (34.4) | 48 (33.8) |
| Time from diagnosis to initial treatment, months, mean (SD) | 30.7 (20.6) | 31.1 (20.6) | 31.3 (20.8) |
| Provider specialty, n (%) |  |  |  |
| Neurology | 97 (56.4) | 96 (53.3) | 70 (49.3) |
| Family medicine | 27 (15.7) | 28 (15.6) | 16 (11.3) |
| Psychiatry | 10 (5.8) | 12 (6.7) | 8 (5.6) |
| Nurse/PA | 8 (4.7) | 9 (5.0) | 7 (4.9) |
| Physical medicine | 0 | 0 | 2 (1.4) |
| Anesthesiology/pain management | 3 (1.7) | 0 | 1 (0.7) |
| Headache orofacial Pain | 4 (2.3) | 3 (1.7) | 2 (1.4) |
| Other^a^ | 23 (13.4) | 34 (18.9) | 37 (26.1) |
| Quan-Charlson comorbidity index score, mean (SD) | 0.63 (1.15) | 0.49 (0.93) | 0.92 (1.32) |
| 0, n (%) | 110 (64.0) | 124 (68.9) | 72 (50.7) |
| 1-3 | 57 (33.1) | 53 (29.4) | 61 (43.0) |
| 4-6 | 4 (2.3) | 3 (1.7) | 8 (5.6) |
| >6 | 1 (0.6) | 0 | 1 (0.7) |

SD, standard deviation.

^a^Other includes specialist, allergy/immunology, other, or missing.
